# Tallapoosa as a Health Resort

**Published:** 1896-08

**Authors:** E. H. Holbrook

**Affiliations:** Tallapoosa, Ga.


### TALLAPOOSA AS A HEALTH RESORT.

TALLAPOOSA, GA., June 28th, 1896.

EDITOR OF THE HOMŒOPATHIC PHYSICIAN.

I am now located in the above place. Please send my Journal there in future.

This is a winter health resort, and a great many have come for their health and remained permanently. The climate here is very fine, indeed. During the past few days, which the old residents say have been very warm, I did not suffer half as much as I have suffered in Baltimore during the hot days of summer. The nights are generally pleasant. The winters are short, and never extremely cold. So there is neither extreme heat in summer nor cold in winter, which makes it very desirable for those who wish to avoid the two extremes, to which more Northern climes are subject.

If you have any patients who come South in winter you cannot do better than recommend them to this city. It is located in the northwestern part of the State, directly west from Atlanta, about two hours' ride from that city. It is almost on the border of Alabama, and is said to have a population of three thousand inhabitants. We have two good hotels here—the Tallapoosa House, which is quite near the depot, and the Lithia Springs Hotel, about half a mile north. Board can be had at private houses. I shall be glad to look after any patients you may send here during the fall and winter.

With kind wishes, yours very truly,

E. H. HOLBROOK, M. D.

